# Nurses’ competence in hemodynamic monitoring and Its impact on clinical decision-making in cardiac ICUs

**DOI:** 10.3389/fcvm.2026.1668297

**Published:** 2026-02-24

**Authors:** Radhwan Hussein Ibrahim, Mariwan Qadir Hamarash, Abdulhakeem Jamil Ahmed, Salwa Hazim Al Mukhtar, Marghoob Hussein Yaas

**Affiliations:** 1 Department of Clinical Nursing Sciences, College of Nursing, Ninevah University, Mosul, Iraq; 2 College of Nursing, AL Kitab University, Kirkuk, Iraq; 3 Department of Nursing, Northern Technical University, Mosul, Iraq; 4 Department of Clinical Nursing Sciences, College of Nursing, University of Mosul, Mosul, Iraq

**Keywords:** clinical decision-making, competence, critical care, hemodynamic monitoring, mixed-methods

## Abstract

**Background:**

Hemodynamic monitoring enables the optimization of care for patients admitted in a critical state. Nurses often rely on their own clinical judgment and intervention in the cardiac ICU environment, especially when feedback loops are interrupted. This is very much dependent on nurses being competent in hemodynamic monitoring and interventions, an area in which research has been limited, particularly in low-resource settings like Iraq.

**Aim:**

The study aimed to investigate the relationships between competencies in hemodynamic monitoring and clinical decision-making, surrounding contextual factors that helped or hindered these practices within the Iraq context.

**Methods:**

To achieve the aim of this study, a mixed-methods approach was used to bring together a cross-sectional survey of the 120 ICU nurses and 17 detailed interviews. The quantitative surveys included during the study produced data with measures of knowledge, interpretation, and clinical decision-making ability. To understand the lived experiences of nurses in this context, qualitative data was collected and systematically analysed using thematic analysis.

**Results:**

The quantitative results found a statistically significant that the competency levels of the hemodynamic monitoring of patients were positively correlated to the quality of clinical decision-making outcomes (*r* = 0.59, *p* < 0.001). The regression analysis found that competency level, years of ICU experience and level of education were significant predictors (*R*^2^ = 0.42, *p* < 0.001). The qualitative analysis identified many barriers to competence, including the absence of advanced training opportunities, relying on physicians for decision-making, and inconsistency of protocols.

**Conclusion:**

Although clinical competence significantly influences nurses’ clinical decision-making, its effective application is strongly shaped by organizational and contextual factors, including training opportunities, workload, and institutional support.

## 1 Introduction

Hemodynamic stability is a cornerstone of life in critical care contexts, particularly in cardiac intensive care units (ICUs) (1). Hemodynamic monitoring is essential for examining a patient's cardiovascular status, including cardiac output, stroke volume, central venous pressure (CVP), mean arterial pressure (MAP), and systemic vascular resistance (SVR) (2). These values serve as indicators to develop and guide fluid therapy, titrate vasoactive medications, and other life-saving interventions (3). Nurses play a pivotal role in monitoring hemodynamic values and are expected to interpret them promptly, leading to informed clinical judgments that can potentially impact patient outcomes (4). Thus, the nurse's ability to perform hemodynamic monitoring is not only a procedural skill, but also an important aspect of clinical judgment and professional role responsibilities (5).

In Iraq, the increasing burden of cardiovascular disease, coupled with the increasing number of ICU admissions, adds to an already pressured healthcare system, further necessitating expert nursing care (6, 7). However, the level of implied competence in the nursing workforce in ICU settings in Iraq may be impacted by limited access to continual professional development, inconsistencies in the quality of nursing education, limited resources in healthcare facilities, and inconsistent use of evidence-based protocols. These hurdles may limit nurses’ abilities when considering hemodynamic data. This may lead to delays in appropriate interventions, resulting in suboptimal care (8, 9).

Prior investigations in developed countries imply an association between nursing competence in hemodynamic skills and nursing outcomes, including decreased ICU mortality and reduced hospital length of stay. There are relatively few studies of this type in low- and middle-income countries (LMIC's) such as Iraq, regarding competency. Consequently, there are specific cultural and contextual factors related to Iraq that should be considered, including constraints in healthcare infrastructure, staffing and availability, the quality of nursing education, economic and resource problems can create special barriers. There is a balance between improvement in the technical aspects of nurses practice, and practicing decision-making and confidence in actualization of hemodynamic monitoring skills, and barriers experienced in practice when trying to apply hemodynamic knowledge at the bedside.

To achieve an overall understanding of nurse competence in hemodynamic monitoring and nurses decision making practice in cardiac ICUs in selected hospitals in Iraq, this study will utilize a mixed-method design. The quantitative aspect will evaluate assessing and utilizing valid survey tools to examine the competence of assessment and skills. The qualitative aspect will look at the nurse's experience of knowledge, barriers, and perception of experience reporting via semi-structured interviews. The mixed-method approach allows for the development of another dimension to understanding competence, and it may also help to identify gaps that can only be identified as blips when using only quantitative analysis.

This study aims to use the findings to help determine sites of possible intervention, direct the focus of nursing education, and to provide preliminary evidence in support of making evidence-based policy in critical care ICU nursing practice. In conclusion, the study is possibly making a contribution to the quality of cardiovascular nursing care, using the findings to enhance patient safety, and enhancing the clinical currency of the nursing workforce in critical and high-acuity clinical practice.

## 2 Materials and methods

### 2.1 Study design

This study employed a convergent parallel mixed-methods design, combining quantitative and qualitative approaches to provide a comprehensive understanding of nurses’ competence in hemodynamic monitoring and its impact on clinical decision-making in cardiac ICUs. Both strands were conducted simultaneously and given equal priority, with integration during the interpretation phase.

### 2.2 Study setting and population

The research was completed in the cardiac intensive care units (ICUs) of three tertiary hospitals in Mosul and Baghdad, Iraq. These sites were selected, as they had high patient volume of cardiac patients and had trained nursing staff to perform care in these units. The population of interest consisted of registered ICU nurses who with at least one year of ICU nursing experience in cardiac intensive care.

### 2.3 Sampling and sample size

Quantitative Phase: A total of 120 ICU nurses were sampled from the population based upon inclusion criteria (Registered Nurses who are employed as full time ICU nurses with at least a one year experience in ICU nursing and willing to take part) and sample size was calculated by means of a power analysis for *α* = .05 and power = .8.

Qualitative Phase: Maximum Variation Sampling was utilized to select a subgroup of 15–20 nurses to be included in the study. The selection process continued until the point of data saturation to ensure that the sample represented a diverse group of nurses in terms of age, years of experience and educational background.

### 2.4 Data collection instruments

#### 2.4.1 Quantitative tools

The quantitative survey was made up of forty-two (42) items and divided among three areas

Theoretical knowledge [fifteen (15) items]

Interpretation of Hemodynamic data [twelve (12) items]

Clinical Application and Practice [fifteen (15) items]

Items were taken from established instruments that have been used to measure hemodynamic data (2, 10). A group of five experts reviewed the instrument to confirm content validity. The Cronbach's alpha was.84, demonstrating high internal reliability. The Clinical Decision Making in Nursing Scale (CDMNS) consisted of forty (40) items, grouped into four (4) dimensions: information gathering, based on evidence, evaluating alternative options and decision making.

#### 2.4.2 Qualitative tools

Interviews were semi-structured in nature to assess how nurses perceived and experienced issues relating to the use of hemodynamic monitoring and decision-making in the intensive care unit (ICU). The semi-structured interviews were based upon an interview schedule; both interviews were audio-recorded and transcribed in their entirety.

A senior academic nurse (holding a doctoral degree) conducted the qualitative interviews as a member of the research team and held formal training in qualitative research methodologies. The researcher conducting the interviews has previous experience with the conduct and analysis of semi-structured interviews in clinical and educational nursing research. The researcher conducting the interviews did not have a direct supervisory, managerial, or evaluative relationship with the participants, thus reducing any potential power imbalance. Before participating in the interviews, all interviewees were provided information about the researcher's role as an academic, the goals of the study, and the voluntary nature of their participation. Interviewees were told the interviews were solely for research purposes and that participant confidentiality would be upheld at all times.

A Doctoral Academic Nurse who is part of the research team conducted the qualitative interviews. She has formally trained in qualitative research methods. This particular researcher has extensive experience in developing and analyzing semi-structured interviews in both educational and clinical nursing research studies. The researcher conducting the interviews did not have a direct supervisor/managerial/evaluation relationship to the participants; she was also not directly involved in their clinical assessment to help limit any possible imbalance in power. All participants were informed prior to the data collection about the researcher's position as an academic, the purpose of the study, and that their participation was voluntary. Participants were also told prior to the interview that the interview was for research purposes only, and that their responses would be kept confidential and would not negatively impact their professional careers.

#### 2.4.3 Qualitative rigour

In order to establish methodological rigour, the following standards established by Lincoln & Guba were adhered to:

##### 2.4.3.1 Credibility

To enhance credibility, full verbatim interview transcripts were returned to a subset of participants for review and confirmation. Member checking was conducted after initial coding and preliminary theme development, enabling participants to verify the accuracy of the transcripts and to comment on the emerging interpretations. Feedback received during this stage was incorporated into the final thematic analysis.

##### 2.4.3.2 Dependability

Maintaining an audit trail, detailing the coding and development of themes.

##### 2.4.3.3 Confirmability

Methodological triangulation was employed to enhance confirmability by integrating findings from both the quantitative and qualitative components of the study. Qualitative themes were systematically compared with quantitative results during the interpretation phase to examine areas of convergence, complementarity, and divergence. This comparative process allowed qualitative findings to contextualize and explain quantitative associations—particularly those related to nurses’ competence and clinical decision-making—thereby strengthening the overall validity and coherence of the mixed-methods conclusions.

##### 2.4.3.4 Transferability

Providing comprehensive descriptions of the study location, participant demographics and the ICU environment to allow comparisons with other environments which are similar.

#### 2.4.4 Interview guide (example questions)

A semi-structured interview schedule was employed in the semi-structured interviews. The semi-structured interview schedule included a series of open-ended questions, such as:

What has been your experience in relation to the use of hemodynamic monitoring equipment?

What difficulties have you experienced in understanding hemodynamic data?

Which of the factors support or impede your decision-making when working in the Cardiac ICU?

How do protocols and teamwork facilitate or inhibit your decision-making in the Cardiac ICU?

Probing questions were utilised as necessary to obtain more detailed and richly descriptive responses.

##### 2.4.4.1 Data collection process

The questionnaires that were completed during the morning shift were returned to the researcher within 24 h of their completion. The semi-structured interviews were carried out in a designated area within the hospital, and lasted around 30–45 min. Anonymity was guaranteed to participants when completing the questionnaires or being interviewed; therefore, a consent form had to be signed prior to both processes (Table 1).

**Table 1:** Timing of qualitative data collection.

Participant ID	Date	Duration (minutes)	Location
P1	Mar 12, 2025	32	Private ICU meeting room
P2	Mar 16, 2025	38	Private ICU meeting room
P3	Mar 21, 2025	41	Private ICU meeting room
…	…	30–45	Same location

Qualitative interviews were recruited using purposeful sampling methods by the researchers in conjunction with unit managers. This ensured diversity in terms of age, clinical experience, and education, as per previously identified inclusion/exclusion criteria. Each interview was performed in a private space with only one participant, and there were never any other individuals present during these interviews. Due to achieving an appropriate level of thematic saturation and sufficient depth in the first interview sessions, repeat interviews were not performed. In addition to recording all responses from the interviews (using audio recordings), the interviewer also kept reflective field notes, documenting contextual information, non-verbal cues and preliminary analytical insights related to the data collected. These were used to assist in interpreting the data during the thematic analysis process.

##### 2.4.4.2 Thematic analysis

Following Braun and Clarke's six step thematic analysis procedure;

Familiarisation,
Initial Coding,
Searching for Themes,
Reviewing Themes,
Defining and Naming Themes,
Producing the Report,

Two researchers coded the data inductively, and disagreements were resolved via dialogue.

The qualitative researchers used an iterative and inductive approach to develop a coding tree as their coding framework, based on the qualitative data collected. The qualitative data management and coding were performed by using NVivo version 14 software, which allowed for the qualitative data to be organized and compared systematically, as well as for the codes across all of the transcripts to be retrieved. After the initial codes were generated, they were grouped together as candidate categories and then reviewed and discussed with the entire research team to either merge or refine these categories as needed. In order to reach agreement on the final themes within the qualitative data, the research team met several times to repeatedly review the qualitative data that had been coded; this process ensured that the themes were clearly distinct, internally consistent and grounded in the participants’ narrative descriptions of their experiences.

The final edited version of the text has been generated to read like it was produced by a person, but all factual information, dates, and material contained in the original text remain unaltered.

### 2.5 Data analysis

The quantitative data were analyzed by utilizing SPSS Version 26. Participant descriptive characteristics were quantitatively analyzed by means of the use of descriptive statistics (i.e., mean, frequency, and standard deviation). Inferentially, Pearson's r and Multiple Regression Analysis were used to determine if there is a relationship between competence and Clinical Decision-Making.

The qualitative data were analyzed using thematic analysis. The transcripts were coded inductively. Themes emerged from a process of iterative coding with a process of constantly comparing the themes throughout the coding process. Member checking, Peer Debriefing, and Audit Trails were utilized to establish trustworthiness.

## 3 Results

### 3.1 Quantitative findings

#### 3.1.1 Demographic characteristics

The total number of ICU nurses that participated in the quantitative phase of this research was 120. The majority of those participants (68.3%) were female, with an average age of 31.6 ± 6.2 years. The vast majority of the participants (81.7%) had a Bachelor's Degree in Nursing, and an average of 4.8 ± 3.1 years of ICU experience (Table 2).

**Table 2:** Demographic and Professional Characteristics of Participants (*N* = 120).

Variable	Category	Frequency (*n*)	Percentage (%)
Gender	Female	82	68.3%
	Male	38	31.7%
Age Group (years)	20–29	36	30.0%
	30–39	58	48.3%
	≥40	26	21.7%
Educational Level	Diploma in nursing	12	10.0%
	Bachelor's degree	98	81.7%
	Master's degree	10	8.3%
ICU Experience (years)	1–3 years	40	33.3%
	4–6 years	48	40.0%
	>6 years	32	26.7%

##### 3.1.1.1 Hemodynamic monitoring skills

Mean competency scores for nurses’ overall proficiency with respect to hemodynamic monitoring ranged from 72.4 ± 9.5, using a 100 point scale. With pre-defined cutoff scores, competency scores were assigned to three categories: Low competency (≤60), Moderate competency (61–79), High competency (≥80). Of the 120 participants, 15.0% (*n* = 18) were classified as low competent, 55.0% (*n* = 66) as moderately competent, and 30.0% (*n* = 36) as highly competent. Nurses’ proficiency scores were highest in areas related to foundational physiological knowledge and lower in the area of interpretation of invasive pressure readings, where there appears to be a deficit in a very important clinical skill which could likely be addressed through targeted education and training (Table 3).

**Table 3:** Levels of Competence in Hemodynamic Monitoring (*N* = 120).

Competence level	Score range	Frequency (*n*)	Percentage (%)
Low competence	≤60	18	15.0%
Moderate competence	61–79	66	55.0%
High competence	≥80	36	30.0%
Mean ± SD	—	—	72.4 ± 9.5

##### 3.1.1.2 Scores on the clinical decision making in nursing scale (CDMNS)

Average scores of 67.2 ± 8.8 on the CDMNS were reported. In addition, it was determined that nurses who have skills related to competency in hemodynamic monitoring have statistically significant higher CDMNS scores (*p* < .01) than those without such skills, suggesting that nurses who are able to measure and understand hemodynamics can also apply evidence based practices in the process of making clinical decisions in the ICUs (Table 4).

**Table 4:** Clinical Decision-Making Scores (CDMNS).

Domain	Mean Score	Standard Deviation
Information Gathering	16.8	3.1
Evidence-Based Reasoning	17.5	2.7
Alternatives Evaluation	16.2	2.9
Final Decision-Making	16.7	3.2
Overall CDMNS Score	67.2	8.8

##### 3.1.1.3 Relationship between competency and clinical decision making

A positive and statistically significant correlation (*r* = 0.59, *p* < 0.001) was found between competency in hemodynamic monitoring and clinical decision making competency, suggesting that as nurses become more skilled at assessing patients’ hemodynamics, they will be more skilled at the clinical decision making process (Table 5). Using multiple regression, the relationship between competency in hemodynamic monitoring and clinical decision making was analyzed, and the results showed that level of competency, years of ICU experience and level of education all predicted how well a nurse performed clinically (*R*^2^ = 0.42, F = 11.6, *p* < .001). This research demonstrates that clinical decision making is complex and multifaceted and that both formal education and clinical experience are required to create value added nursing practice in cardiac ICUs (Table 6).

**Table 5:** Pearson Correlation Between Competence and Decision-Making.

Variables	Correlation coefficient (*r*)	*p*-value
Competence in hemodynamic monitoring	0.59	<0.001**

* *p* < 0.001 is considered statistically significant.

**Table 6:** Multiple Regression Analysis Predicting Clinical Decision-Making Score.

Predictor Variable	B (Unstandardized Coefficient)	β (Beta)	t-value	*p*-value
Competence Score	0.45	0.51	6.43	<0.001**
ICU Experience (years)	0.38	0.24	2.95	0.004**
Educational Qualification	1.12	0.19	2.21	0.029*
Model Summary				
*R*^2^ = 0.42, F = 11.6				<0.001**

* *p* < 0.05, **p < 0.01.

### 3.2 Qualitative findings

#### 3.2.1 Emerging themes

Data analysis of seventeen (17) semi-structured interviews revealed three primary themes and eight (8) sub-themes, which provided insight into how nurses experience their use of hemodynamic monitoring and their clinical decision making (Table 7). The first central theme, “Knowledge Gaps and Learning Needs”, described a broad concern regarding the lack of experience that nurses have with sophisticated monitoring systems. All of the participants reported they lacked sufficient training with respect to interpreting the data from invasive monitoring devices. Additionally, one of the sub-themes that developed included the degree to which nurses and health care professionals relied upon physicians when decisions needed to be made, and thus many times did so with little confidence or familiarity with the hospital's policies.

**Table 7:** Qualitative Themes, Subthemes, and Illustrative Participant Quotes.

Main theme	Subtheme	Illustrative quote
Knowledge gaps and learning needs	Lack of training in advanced monitoring tools	“We often rely on the monitors, but interpreting the numbers correctly requires experience and continuous training, which we rarely receive.”
	Dependence on physicians for decision-making	“Decisions are mostly made by doctors—we observe and follow unless specifically asked for input.”
Barriers in the clinical environment	Work overload and staff shortages	“We are constantly understaffed, which affects how much time we can dedicate to proper monitoring.”
	Limited access to continuing education	“We rarely have access to workshops or updated training on new equipment or protocols.”
	Inconsistent use of protocols	“Different doctors expect different practices—there's no unified guideline in place.”
Professional empowerment and confidence	Gaining confidence through hands-on experience	“With more cases and real-life practice, I've learned to make better judgments over time.”
	Peer learning and informal mentorship	“I learned a lot from my colleagues, especially those who've worked here longer.”
	Desire for autonomy in urgent decision-making	“Sometimes, I want to act quickly without waiting for orders, especially in emergencies.”

The second central theme, “Barriers in Clinical Setting,” identified the system and organizational issues that limit the practical application of nursing. Nurses reported working under extreme workloads and staffing shortages that prevented them from developing the necessary time to acquire new skills and critical thinking abilities to apply to their data. An additional sub-theme was the limited availability of continuing education opportunities for nurses, which indicated there may be additional gaps in continuing education and skill development opportunities for nurses, particularly those employed in public hospitals, that would contribute to an overall lack of skills and knowledge. The variability of the organization's policy implementation between departments and patients created confusion and uncertainty, as well as limited the nurse's autonomy.

The third central theme, “Professional Empowerment and Confidence,” identified the positive experiences that resulted from nurses having more responsibility for their decision making and nursing judgment. Developing clinical skills, possibly more than anything else, was the result of confidence gained through hands-on experiences. Nurses additionally reported that learning from their peers and receiving informal mentoring from colleagues were both important ways of developing competence in real time practice. Additionally, many nurses reported wanting autonomy in urgent clinical decision-making, with many referencing their desire for independence and accountability during emergency situations.

#### 3.2.2 Quotes illustrating important points

Two key quotes from the qualitative study show the two main themes of the study. One quote is:

“Monitors are used all the time. However, it takes experience and continuing education to know what the numbers mean, and both of those resources are rarely available.”

The second quote shows the other theme:

“At times, I am unsure of what to do when I have a patient that has multiple inotropic agents supporting their heart rate at present; however, I have learned to trust my own clinical judgment with continued clinical experience.”

Both of the quotes illustrate the connection between the technological knowledge of nursing practice, the clinical confidence of nursing practice and the importance of a systematic way to develop clinical competence in the nurses.

#### 3.2.3 Quantitative and qualitative data synthesis

*“*Overall, the results of both the quantitative and qualitative findings supported the same conclusion. The quantitative results demonstrated statistical evidence that there is a relationship between nurse capabilities for performing hemodynamic monitoring and making clinical decisions. The qualitative findings allowed for a greater understanding of how other contextual issues may affect the ability to develop clinical competencies/experiences which will lead to developing clinical competence. Overall, the findings of this study demonstrate a clear need for educationally focused programs, access to educational resources, and supportive clinical environments to facilitate the development of clinical competence among cardiac ICU nurses within Iraq.”

## 4 Discussion

This investigation explored the connection between a nurse's competence in the use of hemodynamic monitoring and their clinical decision-making as an ICU nurse caring for cardiac patients in Iraq. A mix-methods design permitted the researcher to conduct a detailed analysis of the relationships between the technical aspects of a nurse's skills; barriers to performance based on the environment; and the cognitive processes that guide a nurse's clinical decisions in a resource-limited environment. The results support the idea that competent hemodynamic monitoring will increase the confidence and efficacy of the clinical decision-making process. In addition, this investigation identified a need for methods of assessing a nurse's competency with hemodynamic monitoring that are based on evidence-based standards or guidelines. These findings were consistent with global studies demonstrating that nurse competency in hemodynamic monitoring is an essential practice for improving patient safety and outcomes (7, 11, 12).

The quantitative findings indicated a moderate level of competence for most nurses, with only 30% attaining high competence—this is consistent with previous research on nurses in low- and middle-income countries, where access to training and advanced equipment are generally constrained (13). the deficits in invasive pressure reading determination suggest a need for specific education to address the identified issues. the large positive correlation between competence and decision-making (*r* = .59, *p* < .001) confirms that to make good decisions in an extremely stressful environment such as a high stakes cardiac ICU setting, a solid conceptual and practical knowledge base of the underlying hemodynamic principles is essential. similarly, ramos et al. (10) found similar results in a brazilian study indicating that competency regarding advanced hemodynamic parameters was associated with improved shock management and reduced lengths of stay in the ICU (10).

This study used multiple regression to determine how much of the variation in nurses’ decision-making performance was due to their competence, years of experience in the intensive care unit (ICU), and level of education. The results showed that competence, years of experience in the ICU, and level of education together accounted for 42 percent of the variation in decision-making performance. Therefore, this study provided empirical support for Benner's “From Novice to Expert” theory. The theory stated that both experiential learning and academic preparation are important components of professional development for nurses. Renner et al. (5), describe repeated exposure to making critical care decisions clinically and receiving explicit instruction in the ICU, allows nurses to develop a pattern recognition system and allow for intuitive judgment skills, similar to those developed by experts.

These findings were supported by qualitative data that revealed additional factors that have hindered nurses’ ability to become competent in decision-making. Structural barriers included, but are not limited to; hospitals lacking adequate resources, continuing professional education being inadequate, and decision-making being primarily physician driven.

These barriers are not unique to Iraq as similar barriers have been reported in studies from loss-affected or resource-limited contexts such as Syria (14), Sudan (15), and some areas of India (16). Lack of simulation-based training experiences and inadequate standardized protocols have been consistently recognized as significant barriers to skills transfer as nurses navigate complex high-stakes environments, like ICUs.

The theme of professional empowerment and confidence resonate with findings from Henao (17) who indicated nurse empowerment and autonomy in clinical decisions tended to not only enhance workplace morale but most importantly, patient outcomes. Participants in this study valued peer learning, mentorship, and experiential learning, which continue to reflect transformational leadership assumptions in nursing education. An astonishing finding in this research was the burgeoning desire for some autonomy by Iraqi nurses when making immediate decisions in urgent patient situations; this is a positive indication of a role getting ready to move forward vs. remain dormant, if systemic kitchen table issues can be addressed.

There is no question about the fact that with these two strands being merged together, we have a powerful message that the evidence is not just to prove that competency exists, but also the Australian Leadership has created an environment where the application of that knowledge can be supported. However, what this looks like may vary depending on the location. Therefore, the main reason for the gap between knowledge and practice is due to institutional barriers as opposed to individuals. As such, we require multi-faceted solutions. Examples of some of these solutions are; investing in nursing education programs, developing national standards or protocols, and mentoring nurses.

There are many international best practices which could be mirrored. For example in Sweden (18) nurses must undertake annual certification for hemodynamic interpretation, while In Australia ICU nurses must have under gone simulation training for every three months (19, 20). Aligning similar continuous learning journey frameworks to an Iraqi context would be beneficial and could yield positive appropriate outcomes. Reinforcing inter- professional collaborative shared-decision-making and teamwork, allow for less-risks to act on patients delays (21–23).

The consequences of these results will be large scale for policy and practice. Nursing leaders in Iraq need to push for structured nursing programs in ICUs that have a large amount of emphasis on hemodynamic monitoring (practical competency, simulation training, and routine assessments). Hospital Administrations need to lessen nurse work loads to provide the extra time to think critically and use evidence based practices. From a larger perspective, it is possible to have a partnership with International organizations to create a model for education to be locally owned and sustainable.

Overall, the results of this study support the relationship between competence in hemodynamic monitoring and the clinical decision-making and decisions that a nurse makes during the care and recovery of a patient in a limited-resource Cardiac ICU. These conclusions from the findings of this study support the urgent need to develop long-term changes at the macro level such as developing new educational systems, empowering the nurses and other staff, and creating institutional support for developing competent and autonomous nurses. Creating a system that addresses the gaps in both skill and structure in the Iraqi health-care system will lead to competent, confident, and autonomous nurses which will lead to better clinical outcomes and safer patients.

The implications for policy and practice are significant. Nursing Leadership in Iraq should support the development of structured Intensive Care Unit (ICU) nursing programs with a high level of emphasis on hemodynamic monitoring, which includes practical competencies, simulation training, and routine assessments. Additionally, hospital administrations must prioritize reducing the workload burdens on nurses to provide them with the necessary bandwidth for critical thinking and evidence-based practice. Furthermore, on a macro level, collaborations with international partners may provide technical expertise, enable the building of local training capacities, and foster the development of sustainable education models.

## 5 Study limitations

There are a number of limitations in relation to the qualitative aspect of the present study that need to be acknowledged. Firstly, while methods to increase rigour were employed, it is unfortunate that reflexivity was not examined in greater detail than this, which could have limited an explicit examination of how the researcher's own influence impacted upon both the process of data collection and data analysis. Secondly, the time frames for the interviews (30–45 min) may have been too short to allow a full exploration of participants’ experiences. Thirdly, the use of purposive sampling of selected cardiac ICUs may produce selection bias and limit inclusion of differing views. Fourthly, due to the contextualised nature of the findings and because the present study focused on cardiac ICUs from selected hospitals in Iraq, there is likely to be significant limitations to the transferability of the qualitative results to different clinical environments or health care systems.

## 6 Conclusion

Clinical competence in hemodynamic monitoring was a major factor contributing to the nurses’ decision making on the basis of this study; however, it became clear from these results that competency by itself is not enough for effective application in the absence of supportive structures at an organizational level. There are structural factors which include workload, access to continuing education, hospital policies/procedures, and degree of professional autonomy that will greatly affect whether or not nurses can effectively apply their competence in real world clinical practice.

### 6.1 Nursing implications

The ramification of our data for practice, education and policy in critical care settings particularly in third world circumstances like Iraq etc., is significant:

1. Structured Training and Skills Development

As proposed in the discussion training and professional development in hemodynamic monitoring and clinical decision making are needed. Nursing curricula need to be amended to provide training in the use of monitoring and decision making tools, particularly in involvement in ICU training modules.

2. Consistency with ICU Protocols

Inconsistency of use of protocol in ICU was a barrier to effective practice. Some agreement on national/institutional level on hemodynamic monitoring and decision making protocol would help eliminate confusion and decrease variation enhancing nurses’ confidence and decisive practice.

3. Creating Culture of Nurse Autonomy and Empowerment

The data demonstrate a tendency for nurses to transfer the lead to be taken by higher authority and a reluctance to take ownership in that role. Leadership and management should foster culture that build trust in nurses to step up as clinical partners taking initiative and responsibility for clinical situation and engage in the clinical decision making.

4. Improving Interprofessional Collaboration

Overall tendency to defer to physician by waiting for directives ultimately results delays in suitable patient management that also marginalizes nurse role. Creating greater opportunities for collaboration and attention to practice models of collaboration with nurses, physicians and other ICU staff can promote better patient care as well as greater esteem for the role of nursing in critical situations.

5. Resource Allocation for ICU Staffing and Training

Healthcare administrators need to appreciate that proper nurse staffing, reduced workload and critical thinking are positively correlated. Investment needs to be made in nurse staffing, equipment etc and time training personnel.

6. Mentoring and Peer-Learning Program

Mentoring and peer-learning was an effective tool. Implementation of mentoring programs for ICU nurses bridge the gap for inexperienced ICU nurses and create an informal collegial similar to tutoring environment.

## Data Availability

The raw data supporting the conclusions of this article will be made available by the authors, without undue reservation.
